# Color Stability of Multi-Layered Zirconia Versus Lithium Disilicate Glass Ceramic: An in Vitro Study

**DOI:** 10.34172/joddd.44008

**Published:** 2026-06-30

**Authors:** Doaa Mohamed Farahat Aboul Azm, Roqaia Mohammad Alassar, Asmaa Ahmed Mosleh, Noureldeen Mohamed Mohamed, Abdullah Waheed Faseeh, Abdelrhman Mohamed Ashour, Farag Rashed Saied

**Affiliations:** ^1^Department of Dental Biomaterials, Faculty of Dentistry, Pharos University in Alexandria, Alexandria, Egypt; ^2^Department of Crowns and Bridges, Faculty of Dental Medicine for Girls, Al-Azhar University, Cairo, Egypt; ^3^Faculty of Dentistry, Pharos University in Alexandria, Alexandria, Egypt; ^4^Department of Conservative Dental Sciences, College of Dentistry, Qassim University, Buraydah, Saudi Arabia; ^5^Department of Operative Dentistry, Faculty of Dentistry for Girls, Al-Azhar University, Cairo, Egypt; ^6^Department of Fixed Prosthodontics, Alexandria University, Former Lecturer of Fixed Prosthodontics, Pharos University in Alexandria, Alexandria, Egypt

**Keywords:** Beverages, Ceramics, Coffee, Color, Immersion, In vitro techniques, Zirconium

## Abstract

**Introduction:**

Patients who receive ceramic restorations use daily oral care products or plaque-control agents, which may cause discoloration of these restorations. The present study assessed the color stability of multi-layered zirconia and lithium disilicate pressable ceramic after 28 days of immersion in chlorhexidine, coffee, and soda.

**Methods:**

A disc measuring 2 mm in thickness and 10 mm in diameter was designed to fabricate 30 discs, divided into two groups based on the ceramic material: multi-layered (ML) zirconia and lithium disilicate (LD). Each group was further subdivided into three subgroups based on the staining solution: chlorhexidine, Cola, and coffee. The samples were immersed in staining liquids at room temperature for 28 days. Color was measured immediately at t_0_ and every week (t_1_, t_2_, t_3_, and t_4_) using a spectrophotometer. Color changes were calculated. For pairwise comparisons, the Friedman test was first employed, followed by post hoc tests.

**Results:**

The greatest ∆E was observed in LD specimens immersed in chlorhexidine (5.65), followed by LD specimens immersed in coffee (5.44). The lowest ∆E was observed for ML zirconia specimens immersed in coffee (0.64). Significant differences were observed between ∆E values of all tested groups and subgroups (*P*≤0.05).

**Conclusion:**

ML zirconia exhibited significantly higher color stability than LD pressable ceramic after being subjected to simulated 28 months of beverage consumption and using chlorhexidine.

**Clinical Significance:**

Multi-layered zirconia exhibited higher resistance to discoloration than lithium disilicate pressable ceramic. Dental professionals should inform patients about the effects of chlorhexidine, Cola, and coffee on lithium disilicate pressable restorations.

## Graphical Abstract

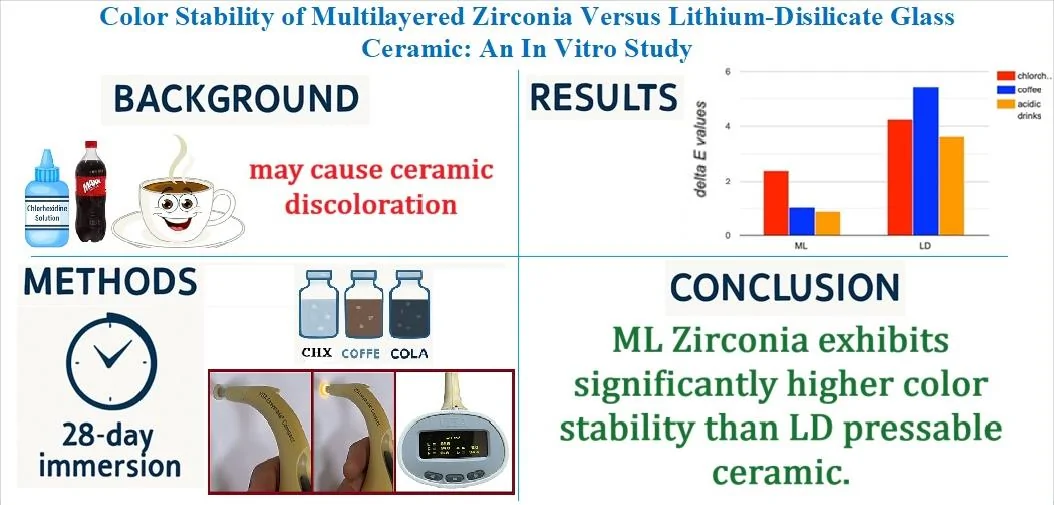

## Introduction

 In contemporary dentistry, the demand for aesthetic restorative materials has driven the development of advanced ceramic systems. Among the materials commonly used in prosthodontics, multi-layered (ML) zirconia and lithium disilicate (LD) glass ceramics have gained widespread acceptance for their favorable mechanical and optical properties.^1^

 Color stability is an essential property influencing the long-term success and patient satisfaction of dental restorations. Regular use of antiplaque mouthrinses or daily drinks is usually recommended for patients with fixed prosthetic appliances. However, these mouthrinses and other drinks may cause discoloration of the ceramic materials. Discoloration can compromise the aesthetics of restorations, necessitating replacement or adjustments over time.^2^

 Monolithic ML Y-TZP zirconia is a polycrystalline ceramic that combines high strength with improved translucency, effectively mimicking the aesthetic effects of dentin and enamel. These materials have gradational chroma and translucency, which encourage their use in anterior and posterior regions. The ML ceramic zirconia has about 43% translucency with variable transmittance among its layers.^3^ On the other hand, LD glass ceramics offer high translucency and a natural enamel-like appearance, along with a proven track record of clinical success. Despite their popularity, the color stability of these materials over time remains an important issue, as food, drinks, and oral hygiene products may contribute to discoloration.^3^

 The color change caused by various beverages in the mouth can be mimicked by immersing a sample material in those drinks in an in vitro model. This method is an effective test for assessing the potential for staining by measuring discoloration levels.^4^ A previous study showed statistically significant discoloration of ML zirconia specimens after their regular immersion in green tea, coffee, and chlorhexidine.^5^ In addition, other investigators revealed that coffee drinks produced the highest discoloration in ML zirconia among all tested beverages.^6^ Previous studies have shown that ML zirconia has many favorable mechanical and aesthetic features; however, its color stability upon exposure to various beverages remains uncertain. Therefore, it is necessary to further examine this restorative material using different beverages and various testing conditions.

 Hence, this study aimed to assess the color stability of ML zirconia and LD pressable ceramic under common discoloration conditions. Clinicians should understand the long-term color stability differences of these materials when selecting the most appropriate option for their patients, ensuring functional durability and aesthetic longevity. The first null hypothesis was that immersion of ML zirconia in chlorhexidine, coffee, and Cola would not affect color stability. The second null hypothesis was that immersion of LD pressable ceramic in chlorhexidine, coffee, and Cola would not affect color stability.

## Methods

###  Sample Size Calculation

 The study was designed to compare the color stability of ML zirconia and LD pressable ceramic. The sample size was calculated using G*Power 3.1.9.7 software. Based on previous studies,^2,7,8^ assuming an alpha error level of 5% and a study power of 80%, a minimum sample size of 30 was needed. Ethical approval was obtained from the Unit of Research Ethics Approval Committee (UREAC), Pharos University in Alexandria (code: PUA-02-2024-12-29-3-309).

###  Specimen Grouping

 Thirty discs were divided into two groups according to the ceramic material: multi-layered (ML) zirconia and lithium disilicate (LD) pressable ceramic. Each group was divided into three subgroups based on the staining solution: chlorhexidine (CHX), coffee, and Cola.

###  Specimen Fabrication

####  Multi-layered (ML) Zirconia

 A disc measuring 2 mm in thickness and 10 mm in diameter was designed using Exocad software (GmbH, Germany) and then sent to a CAD/CAM milling machine (inLab, MC X5, Dentsply, Sirona) for milling 15 ML zirconia discs (shade A2, Cercon ht ML, Dentsply, Sirona) (Figure 1). The milled discs were sintered by firing at 1500ºC for 2 hours and 50 minutes (Nabertherm GmbH, Lilienthal, Germany). The discs were finished with a silicon diamond point (RA3112, Azedent, China) followed by glazing with Cerabien ZR glazing material (Kuraray Noritake Cerabien ZR, Japan) according to the manufacturer’s instructions.

**Figure 1:**
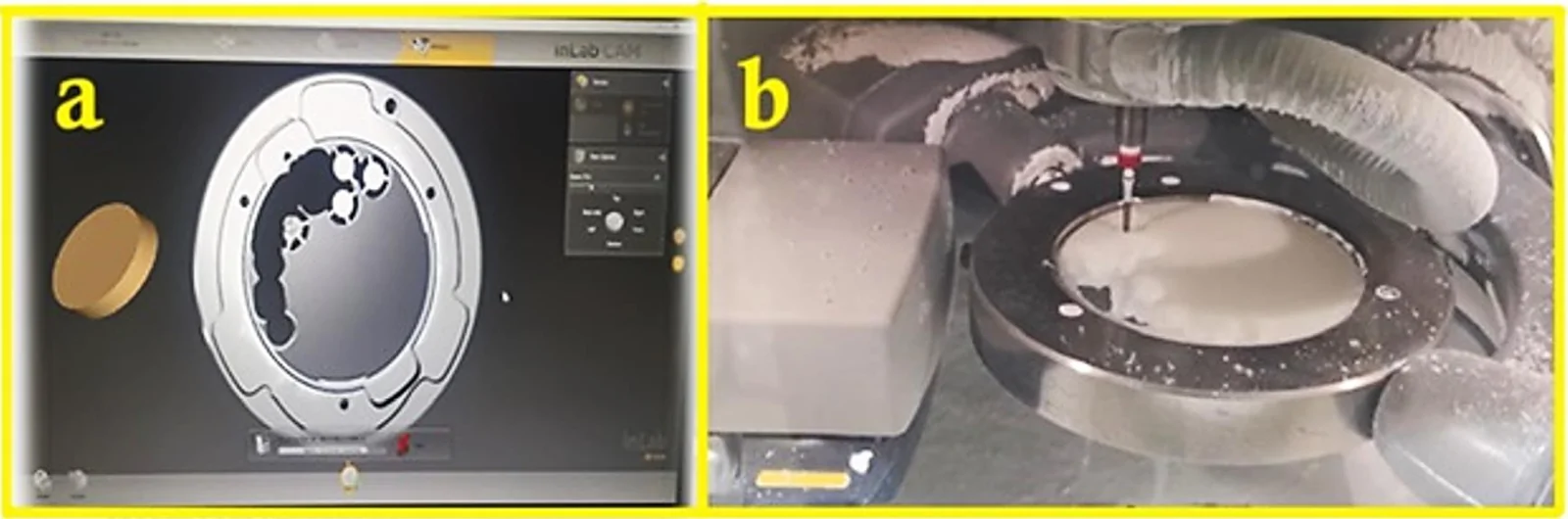
ML zirconia discs fabrication: a) Designing, and b) Milling of ML zirconia discs

####  Lithium Disilicate (LD) Pressable Ceramic

 A disc measuring 2 mm in thickness and 10 mm in diameter was designed using Exocad software (GmbH, Germany) and then sent to a CAD/CAM milling machine (Roland _Dwx-510_, Japan) for milling 15 wax-pattern discs (Dental CAD/CAM Wax-On White disc, On Dent, Germany). The milled wax patterns were sprued (Figure 2), then invested and burned out. The e.max ingots (Shade A2, IPS e.max Press, Ivoclar Vivadent) were heat-pressed in an IPS Empress furnace (Ivoclar Vivadent) at 920ºC, according to the manufacturer’s instructions, to fabricate 15 IPS e.max Press discs. Carbide abrasives (400–1200-grit silicon, Ivoclar Vivadent) were used to finish and flatten the specimens’ surfaces. The discs were cleaned in an ultrasonic cleaner (Darmstadt, Germany) with distilled water for 10 minutes, then glazed with a single layer of IPS e.max ceram glaze(Ivoclar, Schaan, Liechtenstein, Germany) in a Programat CS3 furnace (Ivoclar, Germany) at 710ºC for 6 minutes. Finally, the discs were left to cool at room temperature.

**Figure 2:**
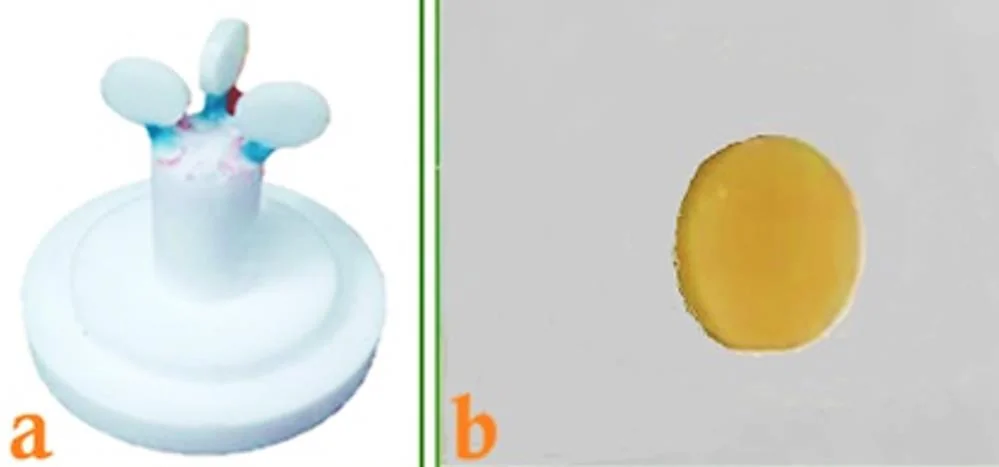
LD pressable ceramic discs fabrication: a) Spruing the milled wax pattern discs, and b) IPS e.max Press disc

###  Staining Process

 The specimens were immersed in the liquids used for staining: chlorhexidine (DG Wash mouthwash, Al Esraa Pharmaceutical Optima, Egypt), coffee (Abu Auf, Turkish Coffee, Egypt), and an acidic drink (MAXI Cola, Egypt) at 20ºC for 28 days. The liquids were refreshed daily. Vials with plastic covers were used to protect the immersion solutions from evaporation.

###  Color Measurements

 After gentle rinsing with distilled water and drying with air (Figure 3), the color of the specimens was measured by a spectrophotometer (Vita Easy Shade Compact, VITA Zahnfabric, Bad Sackingen, Germany) immediately (t_0_) and every week (t_1_, t_2_, t_3_, t_4_) (Figure 4). The color change was calculated using the following formula:

**Figure 3:**
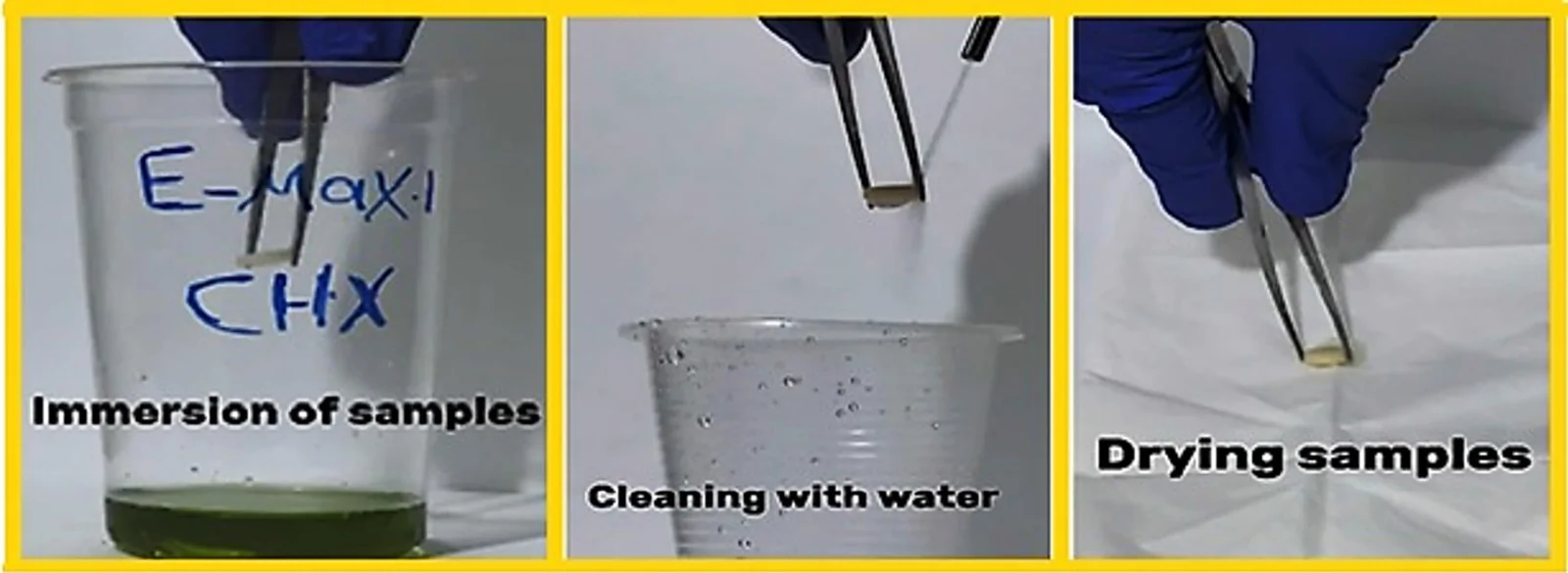
Sample cleaning and drying before testing

**Figure 4:**
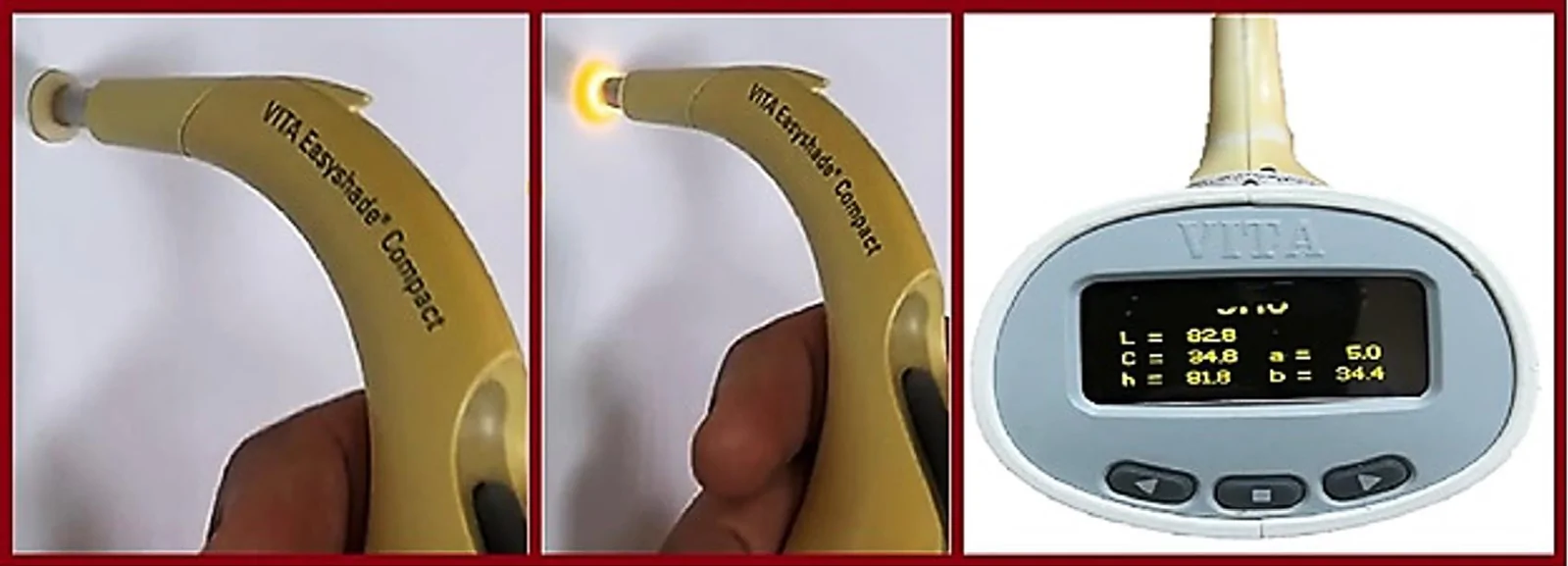
Color measurement using VITA Easyshade

 ΔEab* = [(ΔL*)^2^+(Δa*)^2^+(Δb*)^2^]^1/2^

###  Conversion of ΔE into NBS (National Bureau of Standards) Units9

 The following formula was used to convert the ∆E values to NBS units^9^ as follows:

 NBS Units = ∆E × 0.92

 The use of the National Bureau of Standards units for determining the extent of color change and its correlation with clinical interpretation is summarized and illustrated in Table 1.

**Table 1:** color change interpretation using NBS unit for each case^(9)^

**NBS units**	**Colour Change **	**Clinical Interpretation**
0.00 – 0.50	Traces	Very mild change
0.50 – 1.50	Slightly Noticeable	Mild change
1.50 – 3.00	Noticeable	Perceivable
3.00 – 6.00	Appreciable	Obvious change
6.00 – 12.00	Extreme change	Marked change
> 12.00	Too much	Conversion into another different color

###  Statistical Analysis

 The data were calculated, tabulated, and statistically analyzed using SPSS 25. Friedman’s test was used to compare different follow-up periods, followed by a post hoc test for pairwise comparisons.

## Results

Tables 2 and 3 and Figure 5 show the means and standard deviations (SD) of ∆E values for the studied specimens. The greatest ∆E was observed in LD specimens immersed in chlorhexidine (5.65), followed by LD specimens immersed in coffee (5.44). The lowest ∆E was observed for the ML zirconia specimens immersed in coffee (0.64). Statistical analysis revealed statistically significant differences when the ∆E values of ML zirconia specimens were compared with those of LD specimens, as shown in Table 4 and Figure 5. Significant differences were also observed in the ∆E values between the three subgroups of both materials.

**Table 2:** Δ E values for Multilayered Zirconia specimens

**Interval Time**	**Chlorhexidine** **Mean (SD)**	**Coffee** **Mean (SD)**	**Acidic drink** **Mean (SD)**
T1	1.84(0.034) a	2.00(0.6) a	2.43(0.01) a
T2	2.45(0.34) b	1.17(0.02) a,b	1.53(0.02) a,b
T3	1.91(0.24) a,b	0.64(0.13) b	2.26(0.09) a,b
T4	2.39(0.64) b	1.04(0.02) a,b	0.91(0.01) b
*P* value	0.003*	0.002*	0.002*

*: significance at *P*-value ≤ 0.05. Different manuscript letters indicated significant differences between different follow-up periods. Friedman test was used for comparison between different follow-up periods followed by post hoc test for pairwise comparisons.

**Table 3:** Δ E values for Lithium-disilicate specimens

**Interval Time**	**Chlorhexidine** **Mean (SD)**	**Coffee** **Mean (SD)**	**Acidic drink** **Mean (SD)**
T1	3.69(0.13)a	1.97(0.015)a	2.87(0.02)a,b
T2	5.65(0.02)b	1.94(0.01)a	2.55(0.05)a,b
T3	4.3(0.08)a	2.88(0.04)a,b	2.36(0.04)a
T4	4.26(0.02)a	5.44(0.02)b	3.59(0.06)b
*P* value	0.003*	0.003*	0.002*

*: significance at *P*-value ≤ 0.05. Different manuscript letters indicated significant differences between different follow-up periods. Friedman test was used for comparison between different follow-up periods followed by post hoc test for pairwise comparisons.

**Figure 5:**
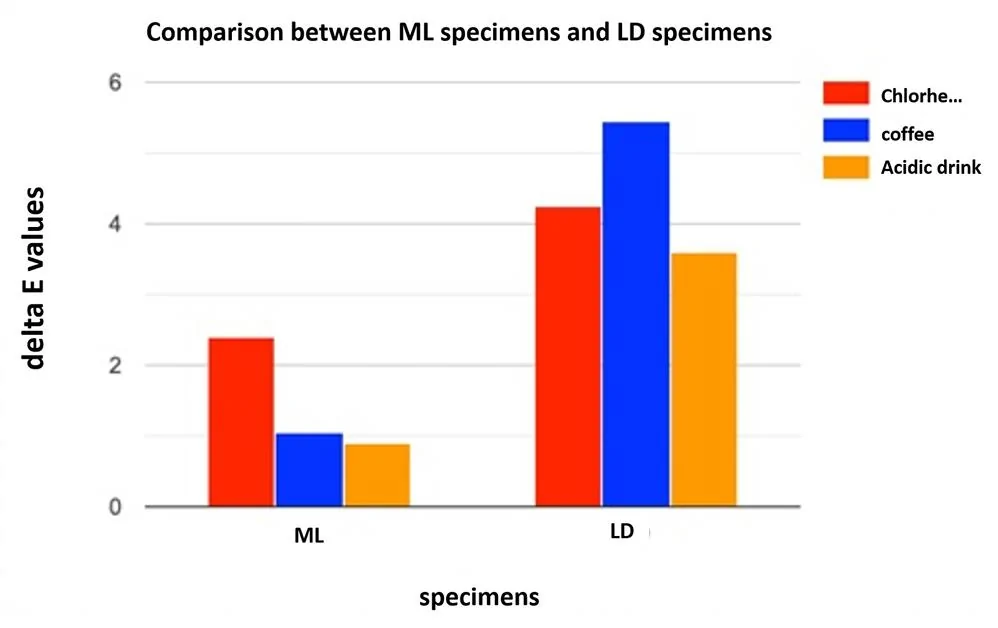
Comparison between the studied groups after 28 days (T4)

**Table 4:** Comparison between ML zirconia and LD pressable ceramic after 28 days

**Groups**	**Chlorhexidine Mean (SD)**	**Coffee** **Mean (SD)**	**Acidic drink** **Mean (SD)**
Multilayered Zirconia	2.39(0.64)	1.04(0.02)	0.91(0.01)
Lithium-disilicate	4.26(0.02)	5.44(0.02)	3.59(0.06)
*P* value	0.008*	0.008*	0.008*

*: significance at *P*-value ≤ 0.05. Different manuscript letters indicated significant differences between different follow-up periods. Mann-Whitney U test was used for comparison between ML zirconia and LD at T4

 The use of the National Bureau of Standards units in determining the extent of color change and its correlation with clinical interpretation is summarized and illustrated in Table 5.

**Table 5:** Color change in ML zirconia and LD after 28 days

**Staining Liquids**	**Groups**	**Delta E values**	**NBS units**	**Clinical Interpretation**
Chlorhexidine	MLZ	2.39	2.2	Perceivable
	LD	4.26	3.9	Obvious change
Coffee	MLZ	1.04	1.0	Mild change
	LD	5.44	5.0	Obvious change
Cola	MLZ	0.91	0.8	Mild change
	LD	3.59	3.3	Obvious change

## Discussion

 The color stability of ceramic materials is a crucial optical characteristic for assessing the success of restorative and prosthetic treatments.^10^ Several factors affect the color of dental ceramics: ceramic type, crystalline content, thickness, surface crack, roughness, sintering method, extrinsic coloring or ceramic stain, glazing material and technique, firing cycles, aging, and surface treatment after artificial aging.^11-17^

 Nowadays, there is a widespread consumption of coffee and Cola despite their ability to discolor teeth and dental restorative materials. In addition, mouthwashes containing chlorhexidine can also cause noticeable discoloration for them.^5^

 This study aimed to compare the effect of chlorhexidine, coffee, and Cola on the color stability of multilayered (ML) zirconia and lithium disilicate (LD) pressable glass ceramics.

 The specimen thickness was 2 mm, similar to the maximum thickness of ceramic restorations.^18^ All the specimens were smoothened, polished, and glazed following the manufacturers’ instructions to avoid surface roughness that causes external staining of surface material.^19^

 The use of glaze seals any ceramic surface defects and provides a glossy, smooth, discoloration-resistant ceramic restoration. A spectrophotometer is an accurate, reliable, repeatable, and convenient color-measuring device.^20^ According to the previously established standards, a ∆E range of 1‒3.3 units represents a considerable difference, but it is clinically acceptable; however, ∆E > 3.3 is perceivable even by an untrained observer and is considered unesthetic and unacceptable.^7^

 The results showed that ML zirconia immersed in chlorhexidine, coffee, and Cola had the lowest ∆E values after 28 days of immersion (Table 4) (Figure 5). Therefore, the first null hypothesis was rejected, as all ML zirconia specimens exhibited perceptible, slight changes in ∆E values when comparing t_0_ and t_4_ (Tables 2 and 5). This finding could be related to five factors.

 First, material composition: Zirconia has a dense crystalline structure with minimal glass content, i.e., less porous than glass ceramics, reducing its tendency to absorb stains from external sources.^21,22^

 Second, layered structure: ML zirconia is engineered with multiple layers of varying translucency and shade properties. This design enhances color stability and aesthetic properties over time, as each layer is tailored to reduce visible discoloration. Khosravani et al.^23^ reported that the layers of ML zirconia affect the final color. In contrast, LD, being a glass ceramic, has a higher degree of translucency and is more prone to color shifts as its glassy matrix can interact with its environment over time.^24^

 Third, resistance to external factors: Zirconia is also more chemically stable than LD, making it less susceptible to acidic or alkaline environments.^25^

 Fourth, glazing: It was necessary to defend against hydrothermal aging of the underlying zirconia.^26^ Researchers recommend polishing and glazing to create a smoother surface and preserve color stability.^16,27,28^

 Fifth, ceramic thickness: The specimen thickness was 2 mm, whereas many studies reported thicknesses of 1 mm, 0.7 mm, and 0.5 mm. The color change was greater in lower ceramic thicknesses.^3,29,30^

 Regarding the effect of chlorhexidine, this study’s findings are consistent with Alzahrani et al.^9^ and Derafshi et al.,^18^ who reported that monolithic zirconia samples immersed in chlorhexidine are susceptible to color changes. However, ∆E values were within clinically acceptable levels.^9,18^ Also, this finding was consistent with Giti et al.,^31^ who demonstrated the influence of chlorhexidine on the color stability of various types of ML zirconia and concluded that ML zirconia is the most color-stable material, with all groups below the clinically acceptable threshold. In the oral cavity, chlorohexidine molecules have been proposed to breakdown into para-chloraniline, which causes denaturation of peptides and production of metal sulfides, which discolor the surfaces of teeth and dental restorations.^32^

 Regarding the effect of coffee, the current findings agree with Abdelhafez et al.^2^ and Elgohary et al.^29^, who reported that coffee affected the color of ML zirconia. However, it remained within the clinically acceptable threshold. ML zirconia showed the greatest color stability, and coffee caused the greatest color change.^2^ In contrast, Colombo et al.^7^ concluded that immersing ML zirconia in coffee-containing solution revealed a statistically significant increase in the values of E. Also, Alzahrani et al.^9^ reported that the greatest color change occurred upon immersion of zirconia in coffee. Coffee has considerable levels of tannins and chlorogenic acid, resulting in significant color changes.^6^ In addition, the acidic pH of coffee could facilitate discoloration.^6^ The coffee pH value generally ranges between 4.85 and 5.10, making it naturally acidic.^33^ Materials immersed in solutions with pH ranging from 4 to 6 have higher sorption values, and the pH of Abo Auf coffee is nearly 5.8, resulting in increased sorption, causing greater change in color.

 The current findings regarding the effect of Cola align with those of Alzahrani et al.^9^ and Abdelhafez et al.,^2^, who noted that zirconia samples exhibited clinically acceptable levels of ∆E after 28 days of immersion in a soft drink. Conversely, Colombo et al.^7^ reported clinically acceptable levels of ∆E after just seven days of zirconia immersion in Cola. The presence of citric acid and synthetic coloring agents in Cola and other acidic beverages can lead to color change of dental restorative materials.^8^ Additionally, the acids in acidic beverages can result in breakdown of metal oxides that eventually leads to peroxide formation, which alters the color of ceramic materials.^34^

 The results showed that LD pressable ceramic immersed in chlorohexidine, coffee, and Cola had the highest ∆E values after 28 days of immersion (Table 4) (Figure 5). Therefore, the second null hypothesis was rejected, as all LD specimens showed marked changes in ∆E values when comparing t_0_ and t_4_ (Tables 3 and 5). The higher color change values for LD specimens than in previous studies may be attributed to different ceramic brands, fabrication techniques, polishing/glazing techniques, and longer immersion times.^1,5,17,35-37^ LD pressable ceramic (IPS e.max press) was tested in this study, and the total immersion time was 28 days, equivalent to 28 months of beverage consumption.^2,8^ According to Palla et al.,^35^ the CAD/CAM lithium disilicate is a more color-stable material than the “press.”

 These findings agreed with those of Sayed et al.,^8^ who concluded that LD exhibited high discoloration, exceeding the clinically acceptable threshold, after being subjected to simulated one-year coffee consumption. In contrast, Haralur et al.,^5^ Sudani et al.,^36^ and Abdelfattah et al.,^17^ reported that LD glass ceramic exhibited clinically acceptable color change after immersion in chlorhexidine, coffee, and acidic drinks, and showed better color stability than monolithic zirconia. According to Mustabshira et al. 1 and a survey of recent literature,^37^ LD ceramic exhibits better color stability and translucency than monolithic zirconia. Palla et al.,^35^ Saleh et al.,^38^ and Esfahanizadeh et al.,^39^ reported LD color change values below the selected acceptability threshold. While Khomprang et al.,^40^ reported no significant difference in ΔE between ML zirconia and LD glass ceramic, both materials showed color change values higher than the acceptable range. All these contradictions may be attributed to methodological differences, as ∆E values typically varied with experimental conditions such as staining liquid brand/concentration, immersion duration/technique, ceramic brand/thickness, sample surface treatments, and glazing material/technique.

 LD ceramics are dense materials composed of crystalline minerals and a glass matrix. However, acidic beverages can cause degradation and ionic disintegration. This process releases alkaline lithium and aluminum ions, which may dissolve the ceramic silicate network.^41^ As a result, the surface may become rougher, allowing for increased staining and discoloration of the ceramic.^7^ Despite its potential limitations, LD seems to be a good material choice with proper case selection, especially given its high esthetic quality. In addition, coffee contains yellow colorants that can cause extrinsic discoloration, leading to significant color changes in LD pressable ceramics.

 The study had several limitations. First, the in vitro design did not accurately reflect real clinical situations, as dental restorations can behave differently when subjected to oral environmental factors such as saliva, toothbrushing, and fluctuating oral pH. Second, the study tested only one brand of each staining liquid, which may limit the generalizability of the findings. A third limitation was the lack of thermomechanical fatigue to observe its potential negative effect on optical properties. Future research should focus on comparing the color change of ceramic dental restorations when subjected to various types of mouth rinses in clinical trials.

## Conclusion

1. ML zirconia has significantly better color stability than LD pressable ceramic after being subjected to simulated 28 months of beverage consumption and using chlorhexidine.
2. ML zirconia showed color changes below the clinically acceptable threshold.
3. LD pressable ceramic showed color changes (> 3) that were clinically unacceptable after 28 days of immersion in chlorhexidine, coffee, and Cola.

## Clinical Significance

1. Multi-layered zirconia exhibited a higher resistance to discoloration than lithium disilicate pressable ceramic.
2. Dentists should warn patients about the effect of chlorhexidine, coffee, and Cola on LD ceramic restorations.

## Competing Interests

 None declared.

## Ethical Approval

 Ethical App Code: PUA-02-2024-12-29-3-309. Unit of Research Ethics Approval Committee Pharos University in Alexandria.
